# Prognostic Significance of Bone Metastasis in Soft Tissue Sarcoma Patients Receiving Palliative Systemic Therapy: An Explorative, Retrospective Pooled Analysis of the EORTC-Soft Tissue and Bone Sarcoma Group (STBSG) Database

**DOI:** 10.1155/2022/5815875

**Published:** 2022-04-01

**Authors:** Georgios Kantidakis, Saskia Litière, Hans Gelderblom, Marta Fiocco, Ian Judson, Winette T. A. van der Graaf, Antoine Italiano, Sandrine Marréaud, Stefan Sleijfer, Gunhild Mechtersheimer, Christina Messiou, Bernd Kasper

**Affiliations:** ^1^EORTC Headquarters, Brussels, Belgium; ^2^Department of Medical Oncology, Leiden University Medical Center, Leiden, Netherlands; ^3^Mathematical Institute Leiden University, Leiden, Netherlands; ^4^Division of Clinical Studies, Institute of Cancer Research, London, UK; ^5^Department of Medical Oncology, Netherlands Cancer Institute, Amsterdam, Netherlands; ^6^Department of Medical Oncology, Erasmus MC Cancer Institute, Erasmus University Medical Center, Rotterdam, Netherlands; ^7^Department of Medical Oncology, Institut Bergonié, Bordeaux, France; ^8^Institute of Pathology, University Hospital Heidelberg, Heidelberg, Germany; ^9^Department of Radiology, The Royal Marsden Hospital, The Institute of Cancer Research, Sutton, UK; ^10^Sarcoma Unit, Mannheim University Medical Center, Mannheim Cancer Center (MCC), University of Heidelberg, Mannheim, Germany

## Abstract

**Background:**

Soft-tissue sarcomas (STS) constitute a rare group of heterogeneous mesenchymal tumours containing more than 100 histologic subtypes. Here, we investigate whether, and if so, to what extent, skeletal metastases affect the outcome of patients with advanced or metastatic disease.

**Materials and Methods:**

Selected patients participated in five clinical trials of EORTC-STBSG. Individuals were included if they started treatment with an active drug and had advanced/metastatic STS. The endpoints of interest were overall survival (OS) and progression-free survival (PFS). Univariate and multivariate pooled analyses (after correcting for 12 covariates) were employed with Kaplan–Meier and Cox regression to model the impact of bone metastasis at presentation per treatment line stratified by study. For the subset of patients with bone metastasis, the impact of another metastatic organ site was explored with multivariate Cox regression models.

**Results:**

565 out of 1034 (54.6%) patients received first-line systemic treatment for locally advanced or metastatic disease. Bone metastases were present in 140 patients (77 first-line, 63 second-line or higher). The unadjusted difference in OS/PFS with or without bone metastasis was statistically significant only for first-line patients. For OS, the adjusted hazard ratios for bone metastasis presence were 1.33 (95%-CI: 0.99–1.78) and 1.11 (95%-CI: 0.81–1.52) for first-line/second-line or higher treated patients, respectively. Likewise, the adjusted hazard ratios for PFS were 1.31 (95%-CI: 1.00–1.73) and 1.07 (95%-CI: 0.80–1.43). Effects were not statistically significant, despite a trend in first-line patients for both endpoints. Subgroup analyses indicated bone and lymph node metastasis as the most detrimental combination for OS and bone and lung metastasis for PFS.

**Conclusions:**

Adult STS patients receiving palliative systemic therapy with bone metastasis carried an overall worse prognosis than STS patients without bone metastases. Skeletal metastasis was detrimental for both OS and PFS, independent of the treatment line. Findings may have implications for the management of these patients.

## 1. Introduction

Soft-tissue sarcomas (STS) constitute a rare group of very heterogeneous mesenchymal tumours that include more than 100 histologic subtypes developed in supportive or connective tissue such as muscle, nerves, blood vessels, and fatty and fibrous tissues [1, 2]. They account for 1-2% of all newly diagnosed malignancies and commonly affect arms, legs, or trunk. Patients with advanced STS have a poor prognosis with a median progression-free survival (PFS) of about 6 months, i.e., for first-line systemic therapy with doxorubicin plus ifosfamide, and median overall survival (OS) of around 12 months.

Some of the most common adult STS histologies are leiomyosarcoma, undifferentiated pleomorphic sarcoma, and liposarcoma [3]. Chemotherapy is the most frequent systemic therapy for unresectable and advanced disease with mostly a palliative intent due to the high percentages of disease progression and mortality. Available treatment options include mostly chemotherapy, for instance, doxorubicin alone or in combination with ifosfamide for the first line and docetaxel plus gemcitabine for second-line or higher treated patients [4, 5]. Pazopanib, a targeted agent, is a treatment option for second-line or higher nonadipogenic STS [5]. The selection of treatment is based on clinical performance, age, histology, disease biology, patient preferences, and availability of novel treatments and studies [6, 7]. Gastrointestinal stromal tumours (GIST) are considered a separate entity since effective targeted treatment is available [8].

Depending on the histology, the majority of STS metastasise primarily to the lungs [9] and sometimes to the lymph nodes, bones, liver, and brain [10, 11]. Other organs may also be affected depending on the sarcoma entity. Skeletal metastasis is part of the natural history affecting prognosis and quality of life of patients with advanced/metastatic disease as a pathological fracture may occur in 20–30% of them together with other skeletal-related events (hypercalcaemia, spinal cord compression, and need for surgery or palliative radiotherapy for refractory pain) [12]. However, staging for bone metastases is not routine, at least for most STS, as their occurrence at presentation is generally rather low [6, 13]. A higher incidence rate is associated with some STS subtypes such as alveolar soft part sarcoma, myxoid liposarcoma angiosarcoma, and rhabdomyosarcoma [12, 14, 15].

In this article, our aim was to investigate (i) whether bone metastasis at presentation is prognostic for OS or PFS of advanced/metastatic STS patients and (ii) which metastatic organ site has the largest impact for patient's OS/PFS combined with bone metastasis at diagnosis in this database.

## 2. Materials and Methods

### 2.1. Patients

Patients with advanced or metastatic non-GIST STS from five prospective clinical trials of the EORTC-STBSG database were included (enrolment period from April 2003 to June 2015) [16–20]. These studies assessed the following drugs/drug combinations: eribulin [17], pazopanib [16, 18], doxorubicin plus ifosfamide versus doxorubicin [19], or trabectedin versus doxorubicin [20]—for either first-line or second-line or later treated locally advanced or metastatic population. The intended treatment arm was used instead of the administered treatment arm as the latter included four missing values and variables were almost identical. Bone lesions were typically detected as part of computed tomography (CT) scans to measure tumour lesions at baseline. Details on eligibility criteria and outcomes have been published [16–20].

**Figure 1:**
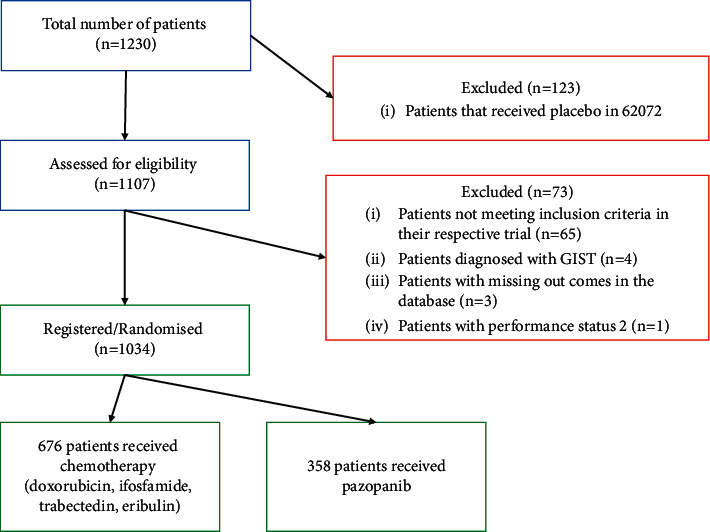
PRISMA flow diagram [21].

Individuals were included if they were eligible in their respective trial, started their allocated treatment with an active drug component, and had locally advanced or metastatic STS at study/observational entry. On the other hand, patients who had a performance status of 2 or worse or were diagnosed with GIST were excluded from all analyses. Data on three patients were not available for both OS and PFS. The PRISMA flow diagram is provided in Figure 1 [21].

In total, 1034 patients were used to investigate the prognostic significance of bone metastasis at study initiation (presence versus absence). A subgroup of 140 patients was analysed to explore the most prognostic metastatic organ site in the concurrent event of bone metastasis.

### 2.2. Endpoints

The endpoints for this analysis were OS and PFS. OS time was estimated from the date of registration/randomisation (according to the study-specific protocol) until the date of death from any cause. Patients still alive were censored on their last follow-up date. PFS time was estimated from the date of registration/randomisation until the date of disease progression or death from any cause. If neither progression nor death was observed, patients were censored on their last date known to be alive.

### 2.3. Adjusted Covariates

Demographic factors at registration/randomisation, line of treatment, histology type, tumour grade, site of the primary tumour, time between histological diagnosis and registration/randomisation, and location of metastasis (bone, liver, lymph nodes, lung, soft tissue (primary or other soft-tissue invasive), or other sites) were considered for the analysis. Soft tissue includes fat, muscles, blood vessels, nerves, tendons, and tissues that surround the bones and joints. Metastasis in other sites referred to ascites, pleural effusion, skin, or other invasive diseases.

Demographic factors included gender, age (less than 40, 40–50, 50–70, or more than 70), and performance status (0 or 1). The line of treatment was 1 (first-line) or 2+ (second-line or higher). The histological entities of STS were aggregated in five commonly occurring groups: angiosarcoma, leiomyosarcoma, liposarcoma (all subtypes), synovial sarcoma, and an additional group for the remaining STS types (other STS). Diagnosis by local pathologists was used as the central review was incomplete for several patients, which could lead to a substantial loss of data.

The tumour grade was dichotomised as low/intermediate or high. Patients whose tumours were initially diagnosed as low grade were only entered in their specific studies in case of rapid progression before first-line systemic therapy as such clinical behaviour is consistent with a higher-grade tumour rather than a low grade. The site of primary tumour was classified into five locations: extremities, abdomen, thorax, visceral, or other (e.g., primary lung is under the thorax; the thigh is under extremities). The time between histological diagnosis and registration/randomisation was categorized as less than a year, 1–2 years, or more than 2 years.

The six covariates for metastasis in bone, liver, lymph nodes, lung, soft tissue (primary or other soft-tissue invasive), or other sites were defined as absent versus present.

### 2.4. Statistical Methods

Covariates were summarised by frequencies and percentages. Univariate analyses were performed for the effect of bone metastasis on OS and PFS per treatment line stratified by study (to account for the variation between the clinical trials) with the Kaplan–Meier method. The log-rank test was used to assess the difference in survival [22]. Moreover, the unadjusted/adjusted effect of bone metastasis on OS and PFS was estimated per treatment line (first-line versus second-line or later) stratified by study with univariate/multivariate Cox regression models, including additionally the baseline variables described in the previous section [23]. For the subset of patients with bone metastasis at presentation, the impact of another metastatic organ site was explored per treatment line with multivariate Cox regression models for PFS and OS stratified by study and adjusted for prognostic variables. The most detrimental combinations are presented.

Outcomes were reported as hazard ratios with 95% confidence intervals (95% CIs). Statistical analyses were performed in SAS software version 9.4 (SAS Institute, Cary NC). All reported *p* values were 2-sided at a 5% significance level.

## 3. Results

### 3.1. Median Follow-Up Times

The median overall follow-up for all patients was 3 years (IQR: 2.2–4.6) estimated with the reverse Kaplan–Meier method [22]. Patients with bone metastasis were followed for up to 5.5 years, whereas those without bone metastasis for a maximum period of 8.0 years. The median overall follow-up time was 2.8 years (IQR: 2.0–5.6) and 3.0 years (IQR: 2.2–4.6) for patients with and without bone metastasis, respectively. Looking at the survivors' group only, the median follow-up time was 2.0 years (IQR: 1.5–2.4) for bone metastasis presence and 2.2 years (IQR: 1.6–3.2) for bone metastasis absence.

### 3.2. Patient Characteristics

In Table 1, baseline characteristics for patients with and without metastasis in the bone are shown. Percentages are similar between the two groups. Systemic therapy was given in first-line to 565 patients (54.6%) with metastatic or locally advanced STS. The majority of the patients (676, 65.4%) received chemotherapy (doxorubicin, doxorubicin plus ifosfamide, eribulin, or trabectedin). Pazopanib (*n* = 358, 34.6%) was the most frequent treatment arm for patients being treated in second-line or higher for metastatic or locally advanced disease. The intended treatment arm and line of treatment in the five EORTC studies are provided in Supplementary Table S1.

The metastatic profile of the patients versus metastasis in bone is shown in Table 2. Bone metastases were present in 140 patients (13.5%); 226 patients (21.9%) had liver metastases, 250 patients (24.2%) had lymph node metastases, and 290 patients (28.0%) had metastases in any other site. On the other hand, pulmonary and soft-tissue metastases (locoregional or other soft-tissue invasive) were present in 719 (69.5%) and 556 patients (53.8%), respectively. Soft tissue metastasis per histology type is provided in Supplementary Table S2.

### 3.3. Prognostic Significance of Bone Metastasis for OS

From the 894 patients without bone metastasis, 488 (54.6%) were in first-line therapy and 406 (45.4%) were treated in second-line or higher systemic treatment for locally advanced or metastatic disease. Amongst the 140 patients with bone metastasis presence at study entry, 77 (55.0%) were treated in the first line and 63 (45.0%) were treated in the second line or later.

**Figure 2:**
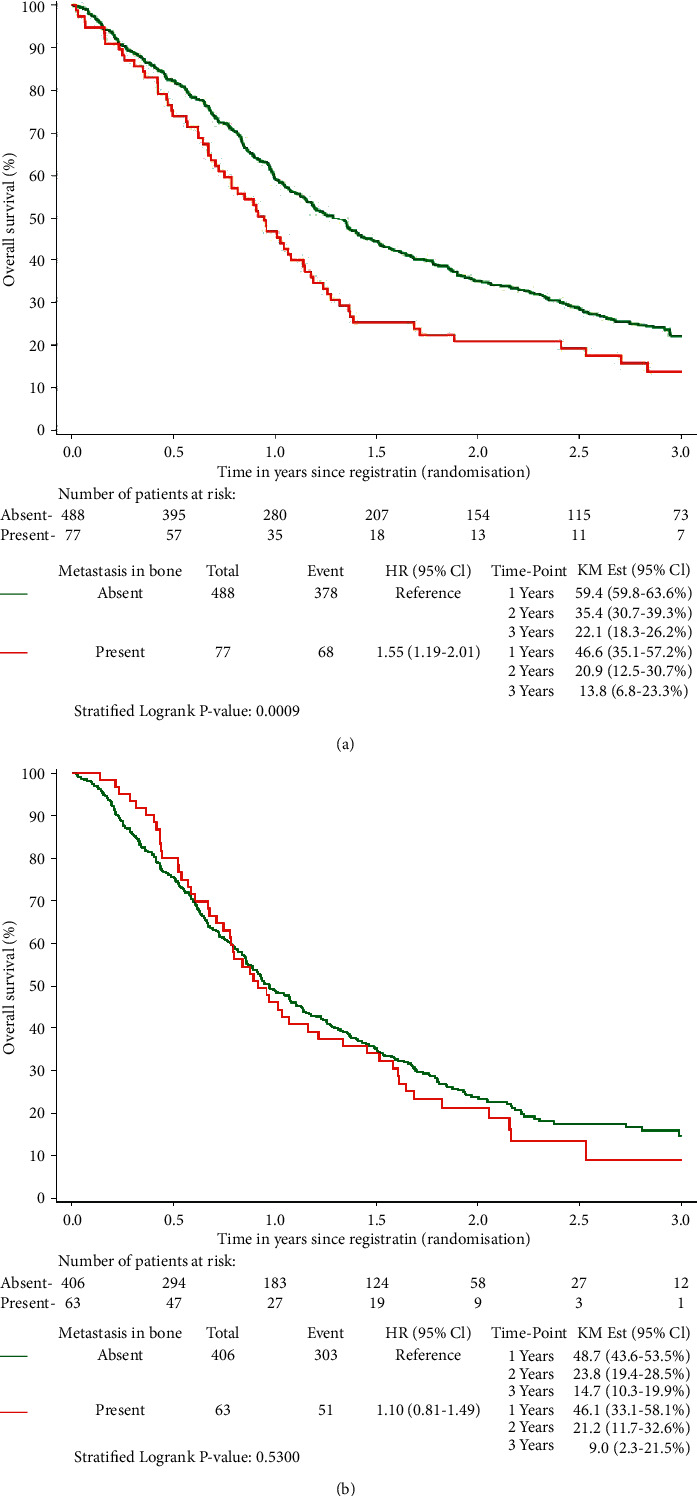
Kaplan–Meier curves for the effect of bone metastasis on OS stratified by study: (a) first line and (b) second line or higher population.

The median first-line OS for patients with or without bone metastases was 0.9 years (95%-CI: 0.7–1.1) and 1.3 years (95%-CI: 1.1–1.4), respectively. For patients treated with second-line or higher systemic treatment, median OS was 0.9 (95%-CI: 0.7–1.2) and 1.0 (95%-CI: 0.9–1.1), respectively. The unadjusted difference in OS for patients with or without metastasis in the bone was statistically significant for first-line (*p* < 0.01) but not for second-line or later systemic treatment (*p*=0.53). Kaplan–Meier curves are presented in Figure 2, including estimates at 1, 2, and 3 years along with their 95%-CIs. The estimated hazard ratio for the presence of bone metastasis was 1.55 (95%-CI: 1.19–2.01) for first-line therapy and 1.10 (95%-CI: 0.81–1.49) for second or further lines of therapy. This means that the presence of bone metastasis increased the hazard of dying by 55.0% for first line. There was no evidence of interaction between bone metastasis and treatment line (*p*=0.13, Figure S1). The adjusted effect of bone metastasis on OS based on multivariate analysis is provided in Table 3. The effect was not statistically significant for any line of treatment (*p* > 0.05)—despite a trend for first-line treatment. The adjusted hazard ratio for bone metastasis presence in first-line systemic treatment was reduced to 1.33 (95% CI: 0.99–1.78). For the population of second line or higher, the adjusted hazard ratio was 1.11 (95% CI: 0.81–1.52).

### 3.4. Prognostic Significance of Bone Metastasis for PFS

**Figure 3:**
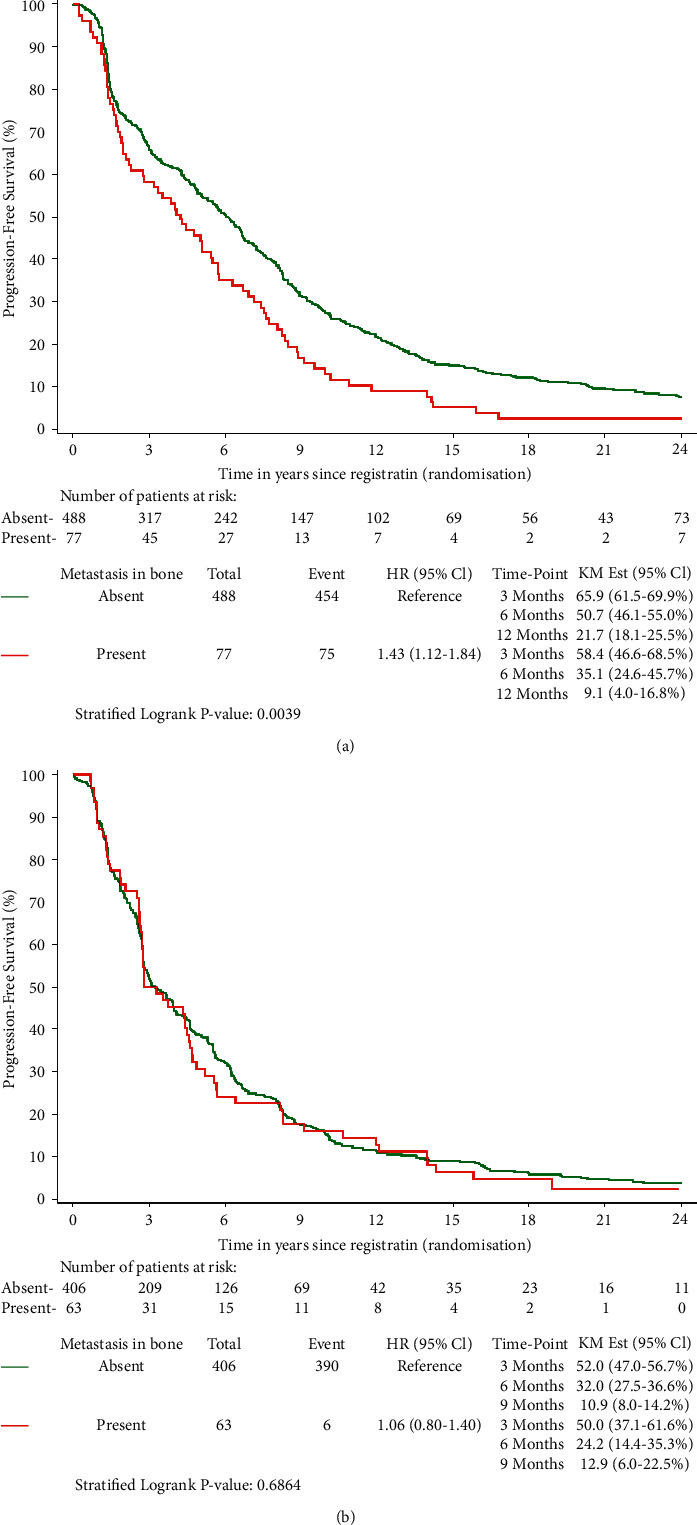
Kaplan–Meier curves for the effect of bone metastasis on PFS stratified by study: (a) first line and (b) second line or higher population.

The median PFS for patients treated in first line was 4.2 (95% CI: 2.3–5.5) and 6.1 months (95% CI: 5.2–6.7) for bone metastasis presence or absence, respectively. For patients treated in second line or higher, the median PFS was 3.0 (95% CI: 2.7–4.6) and 3.3 (95% CI: 2.8–4.0), respectively. The unadjusted difference in PFS for patients with or without metastasis in the bone was statistically significant for first line (*p* < 0.01) but not for second or further lines (*p*=0.69). The corresponding estimated survival curves are presented in Figure 3, including estimates at 3, 6, and 12 months. The unadjusted estimated hazard ratio for the presence of bone metastasis was 1.43 (95% CI: 1.12–1.84) for first line and 1.06 (95% CI: 0.80–1.40) for the population treated in second line or higher for locally advanced or metastatic disease. This means that the presence of bone metastasis increased the hazard of progression or death by 43.0% for the first line. However, there was no evidence of interaction between bone metastasis and treatment line (*p*=0.09, Figure S2).  Table 4 provides the adjusted effect of bone metastasis on PFS based on the multivariate analysis. The effect was not statistically significant for first or further lines (*p* > 0.05)—despite a trend for first line. The adjusted hazard ratio for bone metastasis presence in first line was 1.31 (95%-CI: 1.00–1.73). For population in the second line or higher, the adjusted hazard ratio was 1.07 (95%-CI: 0.80–1.43).

### 3.5. Prognosis for Each Metastatic Organ Site Combined with Bone Metastasis for OS

In our database, 140 patients (13.5%) had bone metastasis and 6 patients had exclusively bone metastasis at presentation. 77 (55.0%) were first-line and 63 (45.0%) second-line or later treated patients. Kaplan–Meier curves for the number of other metastatic organ sites involved together with bone metastasis for OS are depicted in Figure S3 per treatment line. When bone metastasis was present, the number of metastatic organ sites did not seem to affect OS in a proportional manner.

The hazard ratios for the effect of bone metastasis combined with other metastases are presented in Table 5. Bone and lymph node metastasis presence were the most adverse for first-line with a hazard ratio equal to 2.97 (95%-CI: 1.53–5.78). For second-line and higher patients, the combination of bone and lymph nodes seemed to be the most detrimental increasing the risk of death by 59%, although not statistically significant (*p*=0.39).

### 3.6. Prognosis for Each Metastatic Organ Site Combined with Bone Metastasis for PFS

Kaplan–Meier curves for the number of other metastatic organ sites involved with bone metastasis for PFS are provided in Figure S4 per treatment line of systemic treatment. Findings were similar to OS.

The hazard ratios for the combined metastatic profile in the bone and other sites are shown in Table 6. The most detrimental combination was bone and lung metastasis, which increased the hazard of progression or death by 180% in first-line treatment (*p*=0.03) and 145% in second-line or further lines treatment (*p*=0.21).

## 4. Discussion

In this research project, we analysed the prognostic impact of bone metastasis at study inclusion for OS and PFS of locally advanced/metastatic STS, separately for first-line and second-line or higher treated patients, with a pooled analysis of five clinical trials from the EORTC-STBSG database. For the subgroup of patients with bone metastasis (*n* = 140, 13.5%), the most impactful metastatic combination was identified between bone and another site (liver, lymph node, lung, other) for OS and PFS.

There is an increased prevalence of bone metastasis in advanced-stage cancers [24]. The highest incidence can be found in breast, prostate, and lung malignancies [25]. Although bone metastasis is an independent negative prognostic factor with clinical implications for survival and quality of life of patients, a longer survival duration has been observed for breast and prostate cancers with bone metastases, which are hormone-sensitive (median OS 15–27 months) [24–26]. On the other hand, patients with gastrointestinal (GI), lung, and gynaecological cancers usually have the lowest survival in case of bone metastasis (median OS < 12 months). A larger tumour burden is associated with a worse OS.

In general, patients who present with metastatic STS have a poor prognosis regardless of the systemic treatment they receive [27, 28]. A pooled analysis of metastatic STS patients (lesions in lung, liver, bone, or other site)—who received first-line chemotherapy in fifteen EORTC trials—suggested that lung involvement only was an independent prognostic factor in favour of OS in contrast with other metastatic sites [29]. An improved median survival time has also been observed in other studies with isolated lung versus bone metastasis [10, 30].

Ferguson et al. (2006) investigated histologic bone invasion in extremity STS at a reference sarcoma center between 1986 and 2001 based on magnetic resonance imaging (MRI) [31]. In total, 48/874 patients had evidence of bone invasion at presentation. Interestingly, these patients presented with a significantly higher proportion of larger and deeper tumours. They found that bone invasion was a precursor of a poor OS and was associated with a more aggressive clinical course. Younis et al. (2020) used the Surveillance, Epidemiology and End Results (SEER) registry to identify risk factors for early bone metastasis and prognostic factors of survival in 180 extremities of STS patients with skeletal metastasis from 2010 to 2015 [30]. The authors concluded that high tumour grade, deep location to fascia, and regional lymph node metastasis were significant risk factors at diagnosis. Resection of the primary sarcoma was the only significant predictor of survival in the presence of bone metastasis.

A metastatic bone profile may be part of STS patients' natural history, which negatively affects their prognosis. Here, patients with STS of the extremities, abdomen, thorax, visceral or other sites of primary tumour were included. A higher incidence rate of bone metastasis, amongst the four main sarcoma subtypes, was detected for angiosarcoma (10/29, 34.5%) and leiomyosarcoma (46/324, 14.2%), which matches previously reported findings [12, 14]. According to our pooled analysis, the unadjusted difference in OS/PFS for patients with or without bone metastasis was statistically significant for first-line treatment. However, this difference was not significant when adjusting for other prognostic factors. Nevertheless, an overall worse status is suggested for patients suffering from bone metastasis.

A strength of this work is the large patient cohort combined with a minimal amount of missing data. From 1034 patients included here, the tumour grade was missing for 56 patients (5.4%) and the site of primary tumour for two patients (0.2%). The 12 remaining variables were complete, which demonstrates a high-quality data collection in the five EORTC studies. All multivariate Cox models were built adjusting the effects (hazard ratios) for these covariates. In our dataset, 6/1034 patients had exclusively bone metastasis at diagnosis, and therefore a separate analysis of this small subgroup could not be performed. Tentative explanations of this small number could be that (i) bone lesions alone are typically challenging to measure and most trials require a measurable disease to assess response/progression per RECIST 1.1 criteria [32], (ii) bone metastasis at diagnosis is a sign of extensive disease. A limitation of this work is the retrospective exploratory nature. For this analysis, we pooled both randomised and nonrandomised studies from the EORTC-STBSG database to increase the statistical power, which is likely to have introduced some selection bias in the population. Moreover, the interval of follow-up procedures for tumour reevaluation differed between the five trials analysed (e.g., every six or twelve weeks during treatment), which might have had an impact on PFS duration. A subgroup analysis was performed for 140 patients with bone metastasis at presentation to identify the metastatic organ site combination that is the most detrimental for OS and PFS. Due to the limited number of patients per treatment line (77 first-line, 63 second-line or later), results should be interpreted with caution.

Historically, there is heterogeneity in diagnostic tools for bone metastases. Routine use of imaging to detect bone lesions at diagnosis is not standard of care, nor has it ever been, at least for the majority of STS. Most likely, these lesions are detected in a routine computed tomography (CT), which can only detect more advanced bone metastases—e.g., rib or spine metastasis or pelvic disease or when investigating persistent bone pain. The use of more sensitive imaging techniques for screening, such as whole-body MRI and [^18^F]2-fluoro-2-deoxy-D-glucose positron emission tomography/CT (FDG PET/CT), more routinely could increase the detection of bone metastases at diagnosis. However, as FDG PET could also miss bone metastases, whole-body MRI might be a preferable choice (e.g., for myxoid liposarcoma). As patients with metastatic STS survive these days somewhat longer than 20–25 years ago due to advances in supportive and multidisciplinary care, the prevalence of bone invasion is difficult to be ascertained and an increase will inevitably be observed.

According to the latest clinical practice guidelines for diagnosis, treatment, and follow-up of soft-tissue and visceral sarcomas, MRI is the main imaging modality if the primary STS is in the extremities, pelvis, and trunk [5]. Standard X-rays might also help to rule out a bone tumour to detect bone erosion and to show calcifications. When managing patients with advanced/metastatic STS and surgery of lung metastases is selected, it is mandatory to perform an abdominal CT scan and a bone scan or FDG PET to confirm that bone or other lesions are not present. In the case of skeletal metastases, radiotherapy should be considered for palliation of bone lesions at risk of fracture. Orthopaedic intervention is sometimes justified to improve the quality of life of these patients.

## 5. Conclusions

Adult STS patients receiving palliative systemic therapy with bone metastasis demonstrated an overall poor prognosis. A metastatic profile in the bone was detrimental for both OS and PFS in any treatment line, although not statistically significant. The hazard ratios–unadjusted and adjusted—were larger for patients treated in a first-line advanced or metastatic setting. A combined bone/lymph nodes metastatic presentation had the worst OS prognosis. For PFS, bone plus lung metastasis was the most detrimental combination. Of note, such combinations were statistically significant for first-line treatment. These findings may have implications for managing advanced/metastatic STS patients with bone metastasis at diagnosis.

## Figures and Tables

**Table 1:** Patient baseline characteristics versus metastatic profile in the bone.

	Metastasis in bone		
	Absent (*N* = 894)	Present (*N* = 140)	Total (*N* = 1034)
	N (%)	N (%)	N (%)
Intended treatment			
Doxorubicin 75 mg/m^2^	229 (25.6)	32 (22.9)	261 (25.2)
Doxorubicin 75 mg/m^2^/ifosfamide 10 g/m^2^	187 (20.9)	33 (23.6)	220 (21.3)
Pazopanib, 800 mg once daily	309 (34.6)	49 (35.0)	358 (34.6)
Trabectedin 1.3 mg/m^2^, 3 hrs IV	38 (4.3)	6 (4.3)	44 (4.3)
Trabectedin 1.5 mg/m^2^, 24 hrs IV	33 (3.7)	7 (5.0)	40 (3.9)
Eribulin 1.4 mg/m^2^ every 3 weeks	98 (11.0)	13 (9.3)	111 (10.7)
Age category			
Less than 40	194 (21.7)	31 (22.1)	225 (21.8)
40 to 50	201 (22.5)	42 (30.0)	243 (23.5)
50 to 70	428 (47.9)	60 (42.9)	488 (47.2)
Older than 70	71 (7.9)	7 (5.0)	78 (7.5)
Gender			
Male	411 (46.0)	61 (43.6)	472 (45.6)
Female	483 (54.0)	79 (56.4)	562 (54.4)
Tumour grade			
Low/intermediate	411 (46.0)	55 (39.3)	466 (45.1)
High	433 (48.4)	79 (56.4)	512 (49.5)
Missing	50 (5.6)	6 (4.3)	56 (5.4)
Line of treatment			
1st	488 (54.6)	77 (55.0)	565 (54.6)
2+	406 (45.4)	63 (45.0)	469 (45.4)
Site of primary tumour			
Thorax	104 (11.6)	16 (11.4)	120 (11.6)
Abdomen	171 (19.1)	20 (14.3)	191 (18.5)
Extremities	330 (36.9)	54 (38.6)	384 (37.1)
Visceral	194 (21.7)	29 (20.7)	223 (21.6)
Others	94 (10.5)	20 (14.3)	114 (11.0)
Missing	1 (0.1)	1 (0.7)	2 (0.2)
Histology type			
Liposarcoma (all subtypes)	92 (10.3)	8 (5.7)	100 (9.7)
Leiomyosarcoma	278 (31.1)	46 (32.9)	324 (31.3)
Angiosarcoma	19 (2.1)	10 (7.1)	29 (2.8)
Synovial sarcoma	129 (14.4)	13 (9.3)	142 (13.7)
Others	376 (42.1)	63 (45.0)	439 (42.5)
Time between histological diagnosis and registration/randomisation			
Less than a year	377 (42.2)	60 (42.9)	437 (42.3)
1-2 years	174 (19.5)	29 (20.7)	203 (19.6)
More than 2 years	343 (38.4)	51 (36.4)	394 (38.1)
Performance status			
0	494 (55.3)	63 (45.0)	557 (53.9)
1	400 (44.7)	77 (55.0)	477 (46.1)

Treatment regimens in first-line setting: doxorubicin, doxorubicin + ifosfamide, and trabectedin. Treatments regimens for 2^nd^-line or later treated patients: pazopanib and eribulin.

**Table 2:** Patient metastatic profile versus metastasis in the bone.

	Metastasis in bone		
	Absent (*N* = 894)	Present (*N* = 140)	Total (*N* = 1034)
	N (%)	N (%)	N (%)
Metastasis in soft-tissue (primary or other soft-tissue invasive)			
Absent	411 (46.0)	67 (47.9)	478 (46.2)
Present	483 (54.0)	73 (52.1)	556 (53.8)
Metastasis in liver			
Absent	713 (79.8)	95 (67.9)	808 (78.1)
Present	181 (20.2)	45 (32.1)	226 (21.9)
Metastasis in lymph nodes			
Absent	693 (77.5)	91 (65.0)	784 (75.8)
Present	201 (22.5)	49 (35.0)	250 (24.2)
Metastasis in lung			
Absent	292 (32.7)	23 (16.4)	315 (30.5)
Present	602 (67.3)	117 (83.6)	719 (69.5)
Metastasis in other sites (ascites, pleural effusion, skin, or other invasive)			
Absent	649 (72.6)	95 (67.9)	744 (72.0)
Present	245 (27.4)	45 (32.1)	290 (28.0)

**Table 3:** Cox model for the adjusted effect of bone metastasis on OS per line of treatment stratified by study.

Parameter	Levels	Hazard ratio for first-line treatment (95% CI)	*p* value (first-line)	Hazard ratio for second-line or higher treatment (95% CI)	*p* value (second line or higher)
Metastasis in bone	Absent	1.00	0.061	1.00	0.533
	Present	1.33 (0.99, 1.78)		1.11 (0.81, 1.52)	
Histology type (local review)	Angiosarcoma	1.00		1.00	
	Leiomyosarcoma	0.68 (0.39, 1.18)		1.03 (0.40, 2.65)	
	Liposarcoma (all subtypes)	0.65 (0.35, 1.20)		3.05 (1.10, 8.48)	
	Others	0.89 (0.52, 1.54)		2.00 (0.79, 5.04)	
	Synovial sarcoma	0.95 (0.52, 1.73)		2.35 (0.91, 6.04)	
Age category	Less than 40	1.00		1.00	
	40 to 50	1.05 (0.79, 1.41)		1.25 (0.86, 1.81)	
	50 to 70	1.24 (0.93, 1.66)		1.42 (1.05, 1.91)	
	Older than 70	1.25 (0.61, 2.55)		1.88 (1.21, 2.93)	
Gender	Male	1.00		1.00	
	Female	0.95 (0.77, 1.17)		0.89 (0.70, 1.13)	
Tumour grade	Low/intermediate	1.00		1.00	
	High	1.38 (1.11, 1.71)		1.39 (1.11, 1.75)	
Metastasis in liver	Absent	1.00		1.00	
	Present	1.47 (1.11, 1.95)		1.04 (0.79, 1.36)	
Metastasis in lymph nodes	Absent	1.00		1.00	
	Present	1.25 (0.99, 1.56)		1.34 (1.02, 1.77)	
Metastasis in lung	Absent	1.00		1.00	
	Present	1.25 (0.98, 1.59)		1.14 (0.88, 1.49)	
Metastasis in other sites	Absent	1.00		1.00	
	Present	1.53 (1.20, 1.95)		1.36 (1.07, 1.73)	
Metastasis in soft-tissue (primary or other soft-tissue invasive)	Absent	1.00		1.00	
	Present	1.09 (0.86, 1.38)		1.25 (0.98, 1.60)	
Site of primary tumour	Extremities	1.00		1.00	
	Abdomen	1.27 (0.90, 1.78)		1.10 (0.78, 1.54)	
	Others	1.01 (0.73, 1.39)		1.08 (0.72, 1.62)	
	Thorax	1.00 (0.68, 1.47)		1.29 (0.90, 1.85)	
	Visceral	1.21 (0.88, 1.65)		1.25 (0.89, 1.77)	
Performance status	0	1.00		1.00	
	1	1.67 (1.36, 2.05)		1.70 (1.34, 2.17)	
Time between histological diagnosis and registration/randomisation	Less than a year	1.00		1.00	
	1-2 years	0.78 (0.58, 1.06)		0.94 (0.68, 1.30)	
	More than 2 years	0.61 (0.47, 0.79)		0.60 (0.44, 0.80)	

Note that 977 (94.5%) of the patients were analysed due to some missing values.

**Table 4:** Cox model for the adjusted effect of bone metastasis on PFS per line of treatment stratified by study.

Parameter	Levels	Hazard ratio for first-line treatment (95% CI)	*p* value (first-line)	Hazard ratio for second-line or higher treatment (95%-CI)	*p* value (second line or higher)
Metastasis in bone	Absent	1.00	0.054	1.00	0.652
	Present	1.31 (1.00, 1.73)		1.07 (0.80, 1.43)	
Histology type (local review)	Angiosarcoma	1.00		1.00	
	Leiomyosarcoma	1.21 (0.72, 2.04)		0.75 (0.34, 1.67)	
	Liposarcoma (all subtypes)	1.16 (0.66, 2.05)		2.89 (1.15, 7.23)	
	Others	1.33 (0.79, 2.22)		0.81 (0.37, 1.80)	
	Synovial sarcoma	1.39 (0.80, 2.43)		0.85 (0.37, 1.91)	
Age category	Less than 40	1.00		1.00	
	40 to 50	1.10 (0.84, 1.45)		1.00 (0.72, 1.40)	
	50 to 70	0.97 (0.74, 1.27)		1.24 (0.96, 1.61)	
	Older than 70	1.00 (0.53, 1.91)		1.18 (0.80, 1.75)	
Gender	Male	1.00		1.00	
	Female	1.05 (0.86, 1.27)		0.83 (0.67, 1.03)	
Tumour grade	Low/intermediate	1.00		1.00	
	High	1.26 (1.04, 1.53)		1.33 (1.08, 1.63)	
Metastasis in liver	Absent	1.00		1.00	
	Present	1.32 (1.02, 1.72)		1.09 (0.85, 1.39)	
Metastasis in lymph nodes	Absent	1.00		1.00	
	Present	1.13 (0.92, 1.39)		1.18 (0.91, 1.53)	
Metastasis in lung	Absent	1.00		1.00	
	Present	1.32 (1.06, 1.66)		0.93 (0.74, 1.18)	
Metastasis in other site	Absent	1.00		1.00	
	Present	1.42 (1.13, 1.78)		1.36 (1.09, 1.69)	
Metastasis in soft-tissue (primary or other soft-tissue invasive)	Absent	1.00		1.00	
	Present	1.06 (0.85, 1.33)		1.09 (0.87, 1.36)	
Site of primary tumour	Extremities	1.00		1.00	
	Abdomen	1.17 (0.86, 1.60)		0.85 (0.62, 1.15)	
	Others	1.01 (0.75, 1.36)		0.99 (0.69, 1.44)	
	Thorax	1.33 (0.95, 1.87)		1.11 (0.80, 1.55)	
	Visceral	1.21 (0.90, 1.63)		1.24 (0.92, 1.68)	
Performance status	0	1.00		1.00	
	1	1.27 (1.05, 1.54)		1.10 (0.89, 1.36)	
Time between histological diagnosis and registration/randomisation	Less than a year	1.00		1.00	
	1-2 years	0.93 (0.70, 1.23)		1.09 (0.81, 1.47)	
	More than 2 years	0.77 (0.61, 0.97)		0.74 (0.56, 0.96)	

Note that 977 (94.5%) of the patients were analysed due to some missing values.

**Table 5:** Cox model for the effect of bone metastasis combined with another metastatic organ site on OS stratified by study.

Parameter	Levels	Hazard ratio for first-line treatment (95% CI)	*p* value (first line)	Hazard ratio for second-line or higher treatment (95% CI)	*p* value (second line or higher)
Bone and liver metastases	Bone present—liver absent	1.00	0.114	1.00	0.436
	Bone present—liver present	1.83 (0.86, 3.90)		1.56 (0.51, 4.79)	
Bone and lymph node metastases	Bone present—lymph nodes absent	1.00	0.001	1.00	0.389
	Bone present—lymph nodes present	2.97 (1.53, 5.78)		1.59 (0.55, 4.54)	
Bone and lung metastases	Bone present—lung absent	1.00	0.881	1.00	0.915
	Bone present—lung present	0.93 (0.37, 2.36)		0.92 (0.19, 4.47)	
Bone and soft-tissue metastases	Bone present—soft-tissue absent	1.00	0.278	1.00	0.679
	Bone present—soft-tissue present	0.65 (0.30, 1.42)		0.83 (0.34, 2.02)	
Bone and other metastases	Bone present—other absent	1.00	0.044	1.00	0.497
	Bone present—other present	0.45 (0.21, 0.98)		0.68 (0.23, 2.04)	

Hazard ratios were adjusted for demographic characteristics, histological entity, tumour grade, site of primary tumour, and time between histological diagnosis and registration/randomisation.

**Table 6:** Cox model for the effect of bone metastasis combined with another metastatic organ site on PFS stratified by study.

Parameter	Levels	Hazard ratio for first-line treatment (95% CI)	*p* value (first-line)	Hazard ratio for second-line or higher treatment (95% CI)	*p* value (second line or higher)
Bone and liver metastases	Bone present—liver absent	1.00	0.185	1.00	0.149
	Bone present—liver present	1.68 (0.78, 3.63)		2.16 (0.76, 6.15)	
Bone and lymph node metastases	Bone present—lymph nodes absent	1.00	0.040	1.00	0.742
	Bone present—lymph nodes present	1.99 (1.03, 3.85)		0.84 (0.30, 2.34)	
Bone and lung metastases	Bone present—lung absent	1.00	0.030	1.00	0.205
	Bone present—lung present	2.80 (1.10, 7.09)		2.45 (0.61, 9.84)	
Bone and soft-tissue metastases	Bone present—soft-tissue absent	1.00	0.299	1.00	0.175
	Bone present—soft-tissue present	0.68 (0.33, 1.40)		1.94 (0.75, 5.04)	
Bone and other metastases	Bone present—other absent	1.00	0.112	1.00	0.082
	Bone present—other present	0.56 (0.27, 1.15)		0.41 (0.15, 1.12)	

Hazard ratios were adjusted for demographic characteristics, histological entity, tumour grade, site of primary tumour, and time between histological diagnosis and registration/randomisation.

## Data Availability

The data that support the findings of this study are available from 5 clinical trials (62012, 62043, 62052, 62072, 62091) of the European Organisation for Research and Treatment of Cancer-Soft Tissue and Bone Sarcoma Group (EORTC-STBSG) database. Data can be requested via https://www.eortc.org/data-sharing/.

## Supplementary Materials

Supplementary Materials Supplementary Table S1: intended treatment arm and line of treatment per EORTC study. Supplementary Table S2: soft-tissue metastasis (primary or other soft-tissue invasive) per histology type. Supplementary Figures S1-S2: forest plot of the (unadjusted) interaction effect between treatment line and bone metastasis for OS/PFS. Supplementary Figures S3-S4: estimated OS/PFS for the number of other metastatic organ sites involved with bone metastasis: (a) first-line and (b) second line or higher for the locally advanced or metastatic population. .
