# Supplementary material for: Prognostic Significance of Bone Metastasis in Soft Tissue Sarcoma Patients Receiving Palliative Systemic Therapy: An Explorative, Retrospective Pooled Analysis of the EORTC-Soft Tissue and Bone Sarcoma Group (STBSG) Database
Source: Sarcoma. 2022 Apr 1;2022:5815875. doi: 10.1155/2022/5815875 (PMC8993578; doi:10.1155/2022/5815875)
Supplement: Supplementary Materials — Supplementary Table S1: intended treatment arm and line of treatment per EORTC study. Supplementary Table S2: soft-tissue metastasis (primary or other soft-tissue invasive) per histology type. Supplementary Figures S1-S2: forest plot of the (unadjusted) interaction effect between treatment line and bone metastasis for OS/PFS. Supplementary Figures S3-S4: estimated OS/PFS for the number of other metastatic organ sites involved with bone metastasis: (a) first-line and (b) second line or higher for the locally advanced or metastatic population. . [file 5815875.f1.docx]

**Prognostic significance of bone metastasis in soft tissue sarcoma patients receiving palliative systemic therapy: an explorative, retrospective pooled analysis of the EORTC - Soft Tissue and Bone Sarcoma Group (STBSG) database**

Georgios Kantidakis, Saskia Litière, Hans Gelderblom, Marta Fiocco, Ian Judson, Winette T.A. van der Graaf, Antoine Italiano, Sandrine Marréaud, Stefan Sleijfer, Gunhild Mechtersheimer, Christina Messiou, Bernd Kasper

SUPPLEMENTARY MATERIAL

Correspondence to:

Prof Hans Gelderblom,

Department of Medical Oncology,

Leiden University Medical Center, 2333 ZA,

Leiden, Netherlands

[a.j.gelderblom@lumc.nl](mailto:a.j.gelderblom@lumc.nl)

**Table S1: Intended treatment arm and line of treatment per EORTC study.**

|  | **Study** | | | | | **Total (N=1034)** |
| --- | --- | --- | --- | --- | --- | --- |
|  | **62012 (N=439)** | **62043 (N=137)** | **62052 (N=111)** | **62072 (N=221)** | **62091 (N=126)** |  |
|  | **N (%)** | **N (%)** | **N (%)** | **N (%)** | **N (%)** | **N (%)** |
| **Intended treatment** |  |  |  |  |  |  |
| **Doxorubicin 75mg/m²** | 219 (49.9) | 0 (0.0) | 0 (0.0) | 0 (0.0) | 42 (33.3) | 261 (25.2) |
| **Doxorubicin 75mg/m²**  **/ifosfamide 10g/m²** | 220 (50.1) | 0 (0.0) | 0 (0.0) | 0 (0.0) | 0 (0.0) | 220 (21.3) |
| **Pazopanib, 800 mg once daily** | 0 (0.0) | 137 (100.0) | 0 (0.0) | 221 (100.0) | 0 (0.0) | 358 (34.6) |
| **Trabectedin 1.3 mg/m², 3 hrs IV** | 0 (0.0) | 0 (0.0) | 0 (0.0) | 0 (0.0) | 44 (34.9) | 44 (4.3) |
| **Trabectedin 1.5 mg/m², 24 hrs IV** | 0 (0.0) | 0 (0.0) | 0 (0.0) | 0 (0.0) | 40 (31.7) | 40 (3.9) |
| **Eribulin 1.4 mg/m² every 3 weeks** | 0 (0.0) | 0 (0.0) | 111 (100.0) | 0 (0.0) | 0 (0.0) | 111 (10.7) |
| **Line of treatment** |  |  |  |  |  |  |
| **1** | 427 (97.3) | 2 (1.5) | 0 (0.0) | 13 (5.9) | 123 (97.6) | 565 (54.6) |
| **2+** | 12 (2.7) | 135 (98.5) | 111 (100.0) | 208 (94.1) | 3 (2.4) | 469 (45.4) |

**Table S2: Soft-tissue metastasis (primary or other soft-tissue invasive) per histology type.**

|  | **Liposarcoma (N=100)** | **Leiomyosarcoma (N=324)** | **Angiosarcoma (N=29)** | **Synovial sarcoma (N=142)** | **Other (N=439)** | **Total (N=1034)** |
| --- | --- | --- | --- | --- | --- | --- |
|  | **N (%)** | **N (%)** | **N (%)** | **N (%)** | **N (%)** | **N (%)** |
| **Metastasis in soft-tissue** |  |  |  |  |  |  |
| **Absent** | 19 (19.0) | 169 (52.2) | 6 (20.7) | 85 (59.9) | 199 (45.3) | 478 (46.2) |
| **Present** | 81 (81.0) | 155 (47.8) | 23 (79.3) | 57 (40.1) | 240 (54.7) | 556 (53.8) |

**
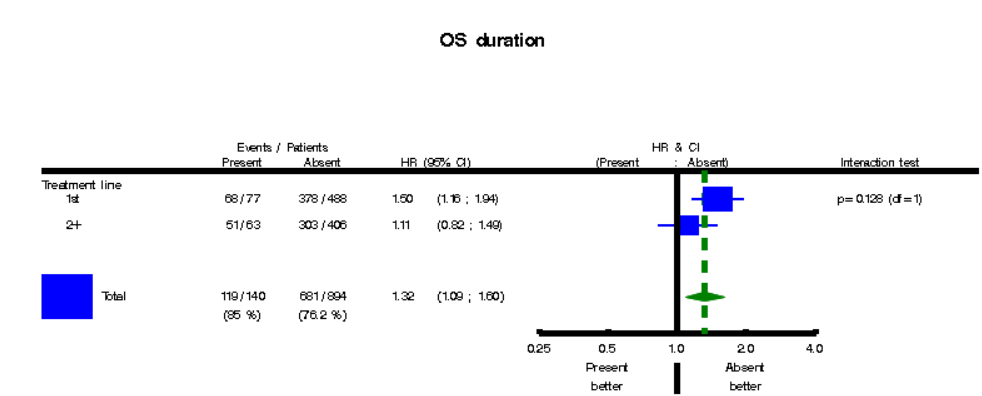
**

**Figure S1: Forest plot of the (unadjusted) interaction effect between treatment line and bone metastasis for OS.**

***
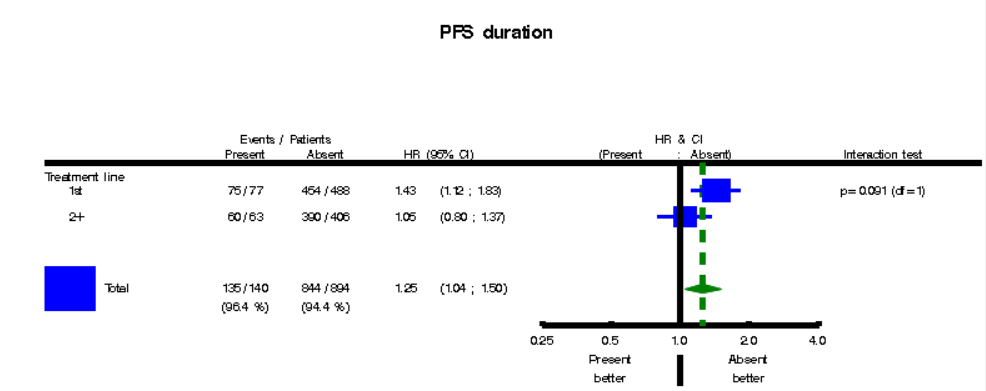
***

**Figure S2: Forest plot of the (unadjusted) interaction effect between treatment line and bone metastasis for PFS.**


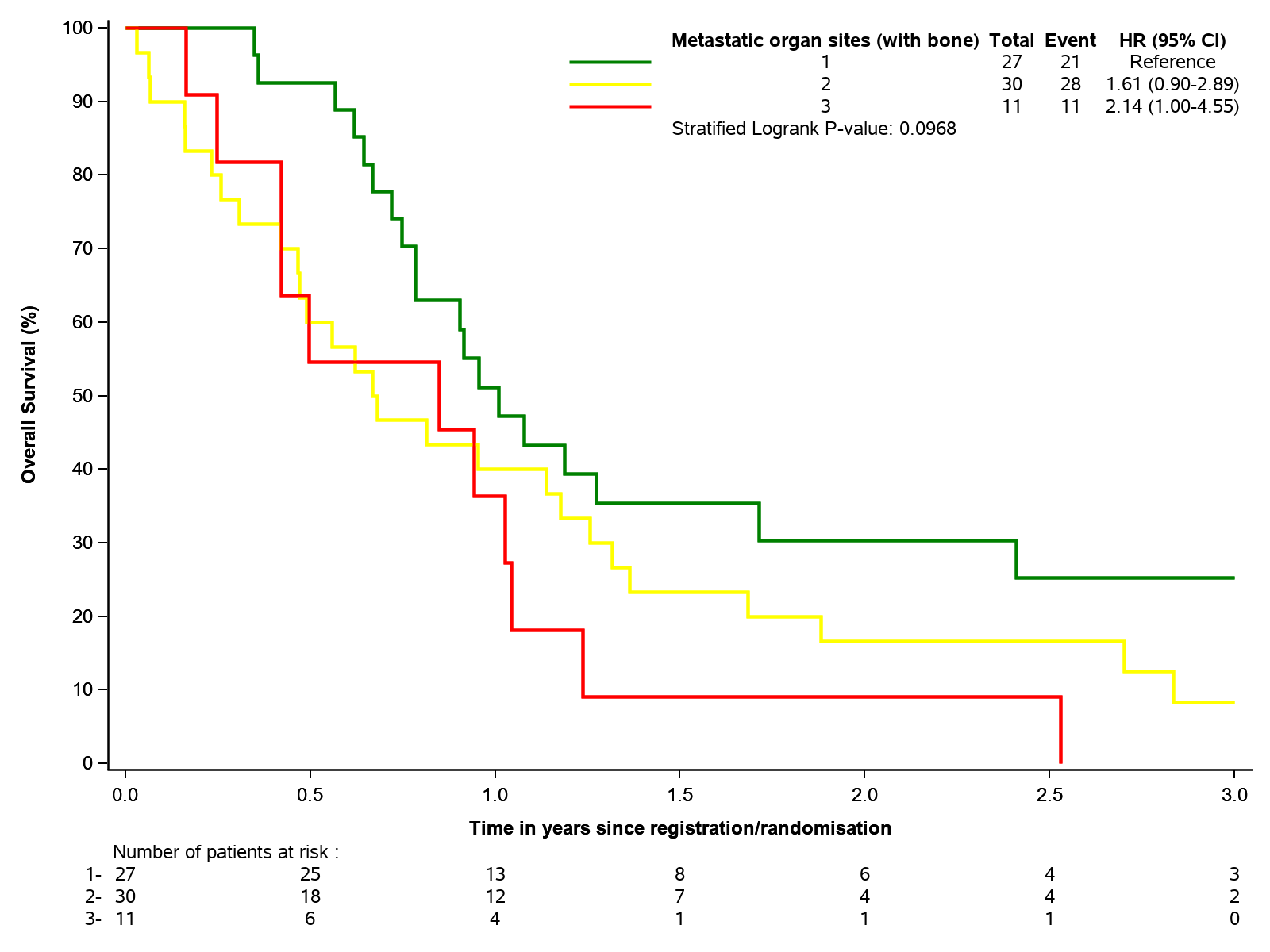


a


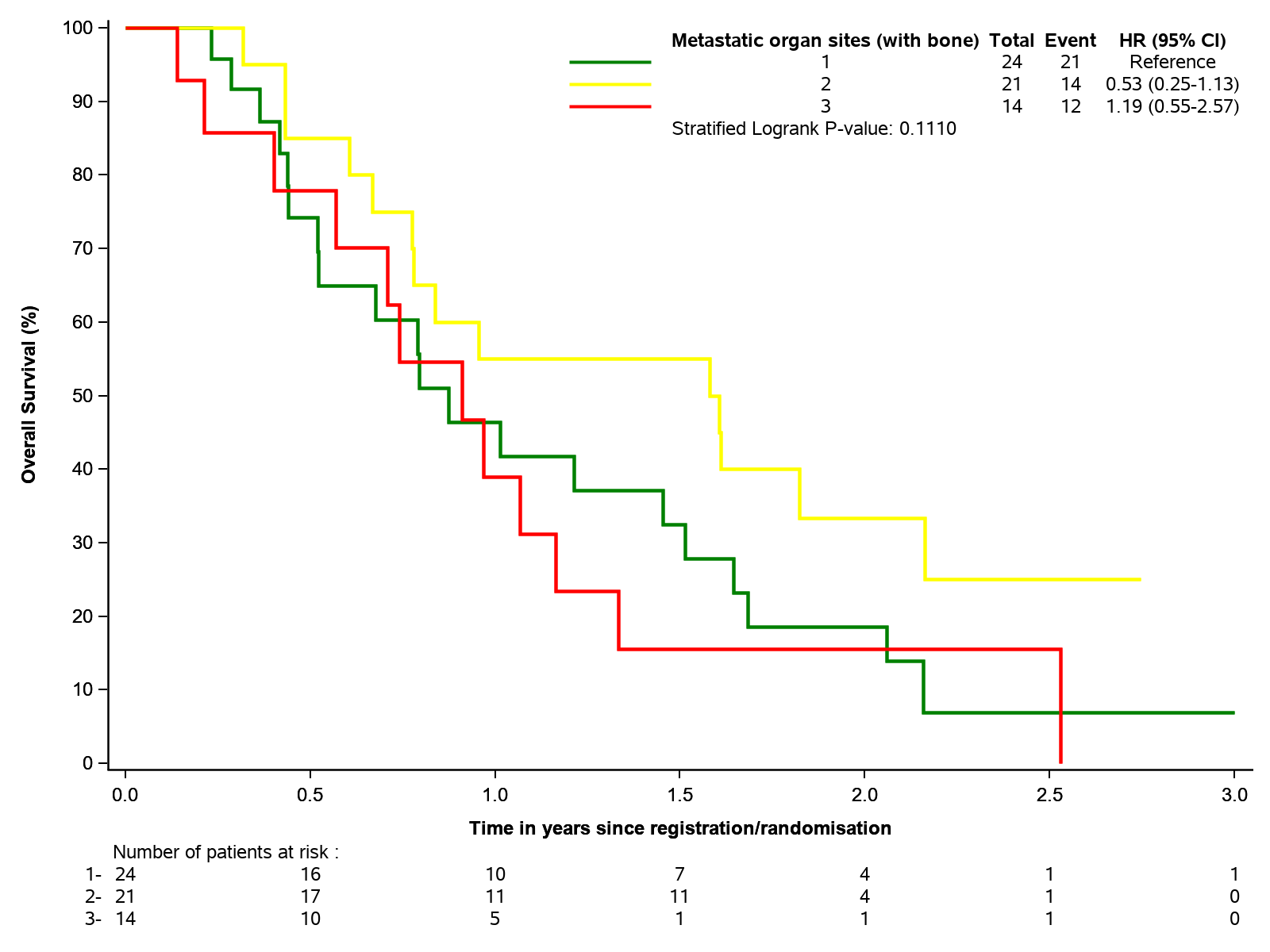


b

**Figure S3: Estimated OS for the number of other metastatic organ sites involved with bone metastasis; (a) first-line, (b) second line or higher for locally advanced or metastatic population.** This subgroup analysis included 77 first-line and 63 second line or higher treated patients with bone metastasis. Sites for possible metastases included liver, lymph nodes, lung and other (ascites, pleural effusion, skin or other invasive). Note that the lines for 0 (bone metastasis only) and 4 metastases were removed as they included only 6 (first-line) and 7 (3 first-line, 4 second line or higher) patients, respectively.


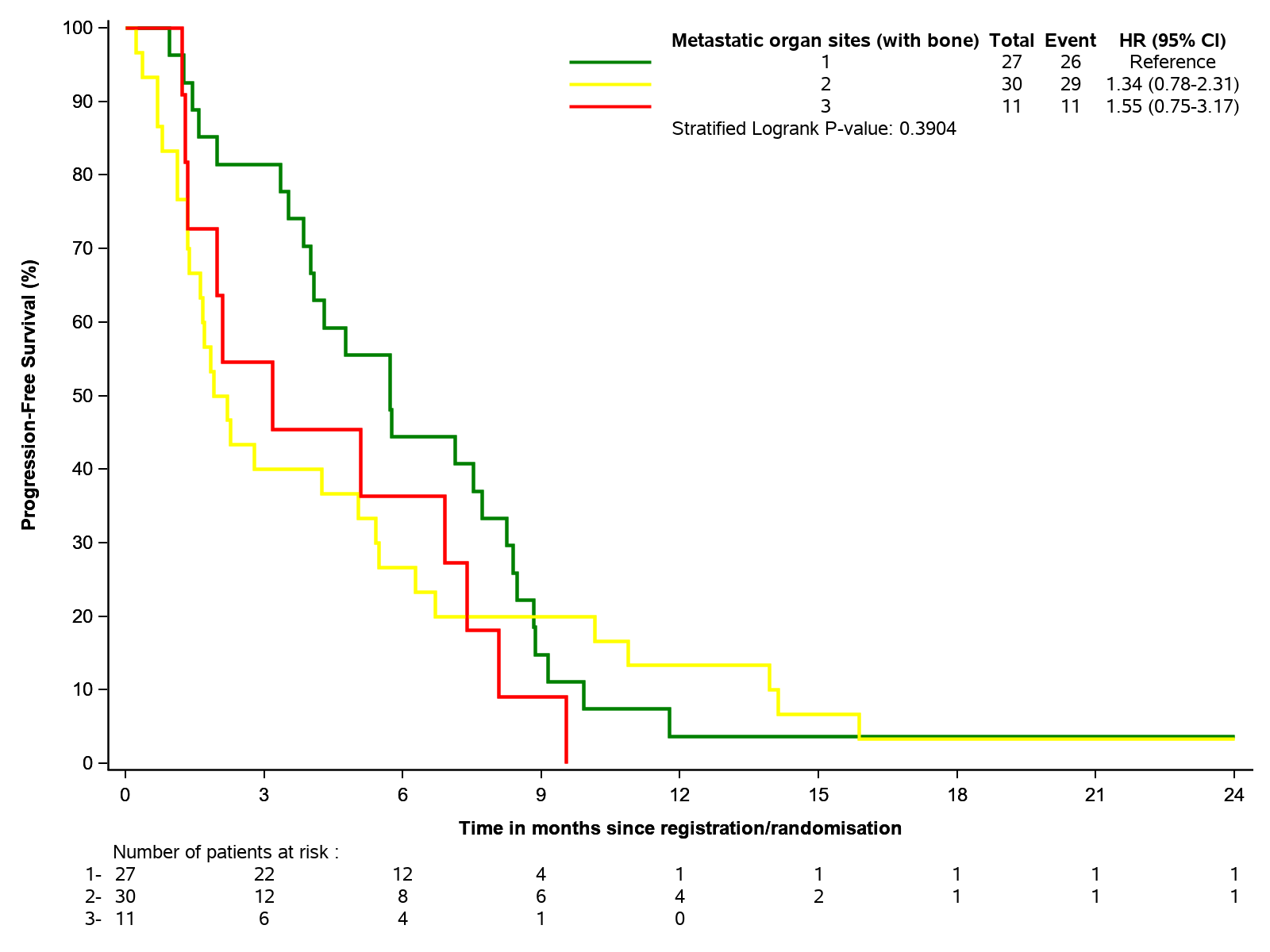


a


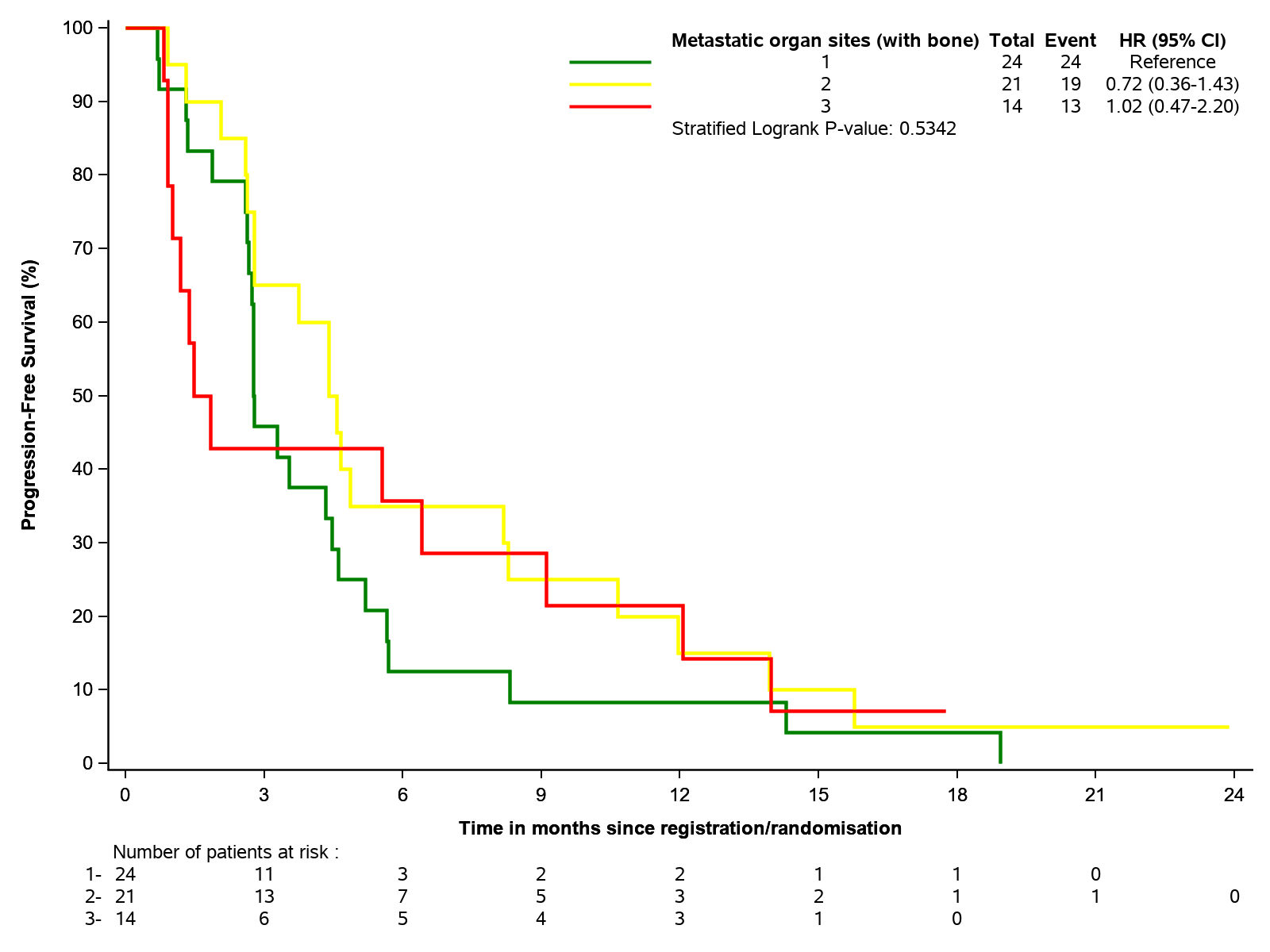


b

**Figure S4:** **Estimated PFS for the number of other metastatic organ sites involved with bone metastasis; (a) first-line, (b) second line or higher for locally advanced or metastatic population.**
